# Left Upper Lobe Lung Cancer With Swyer-James-MacLeod Syndrome

**DOI:** 10.1016/j.atssr.2025.02.012

**Published:** 2025-03-10

**Authors:** Motoka Omata, Shota Mitsuboshi, Hiroaki Shidei, Akira Ogihara, Hiroe Aoshima, Tamami Isaka, Takako Matsumoto, Masato Kanzaki

**Affiliations:** 1 The Department of Thoracic Surgery, Tokyo Women’s Medical University, Tokyo, Japan; 2 Integrated Education Center, Tokyo Women’s Medical University, Tokyo, Japan

## Abstract

Swyer-James-MacLeod syndrome (SJMS) is characterized by unilateral hyperlucency of the lung on chest roentgenogram, unilateral reduction in vascularity on chest computed tomography, and unilateral loss of perfusion on pulmonary ventilation/perfusion scan. The prevalence of SJMS is reported to be 0.01%, with most cases occurring in children and only a few cases in adults. Therefore, SJMS associated with primary lung cancer is extremely rare. We present a case of left upper lobe lung cancer with SJMS by performing robot-assisted thoracoscopic left pneumonectomy.

Swyer-James-MacLeod syndrome (SJMS) is a rare disease, in 1953 first reported by Swyer and James^1^ in cases in children, and in 1954 by MacLeod^2^ in adults. It is characterized by unilateral hyperlucency of the lung on chest roentgenogram, unilateral reduction in vascularity on chest computed tomography (CT), and unilateral loss of perfusion on pulmonary ventilation/perfusion scan.^3^

SJMS associated with primary lung cancer is extremely rare, with only a few cases reported.^4^ Here, we report a case of left upper lobe lung cancer associated with SJMS in which left pneumonectomy was performed using robotic surgical system.

A man in his 70s visited the hospital complaining of chest pain. A chest CT showed a 50-mm mass in the left upper lobe. The specimen from a transbronchial lung biopsy of the tumor yielded a diagnosis of squamous cell carcinoma. He was then referred to our hospital for surgery.

Physical examination revealed no abnormalities. Tumor markers were detected as the following: squamous cell carcinoma antigen, 4.8 ng/mL (normal range, ≤1.5 ng/mL); and cytokeratin-19 fragment, 5.1 ng/mL (normal range, <3.3 ng/mL). Pulmonary function test result demonstrated forced expiratory volume in 1 second of 1.57 liters (52.5% predicted) and forced expiratory volume in 1 second/forced vital capacity ratio of 54.5%. Echocardiography showed fractional shortening of 0.43, ejection fraction of 0.60, and no abnormal findings.

Chest roentgenogram revealed a mass shadow in the left upper lung field (Figure 1), chest CT showed an irregular mass, 53 mm in diameter, in the left upper lobe, and both hyperlucency and diminished vascularity in the left lower lobe (Figures 2A, 2B). A 3-dimensional CT also showed hypoplasia of the left pulmonary artery (A6-10) and an inferior pulmonary vein (Figure 2C)

**Figure 1:**
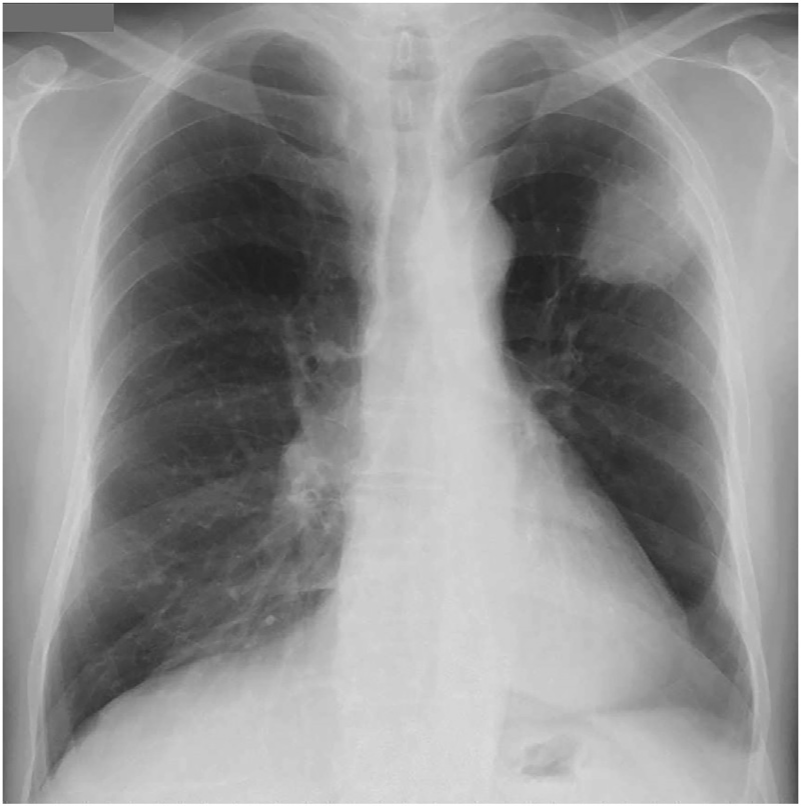
Chest roentgenogram revealed a mass shadow in the left upper lung field.

**Figure 2:**
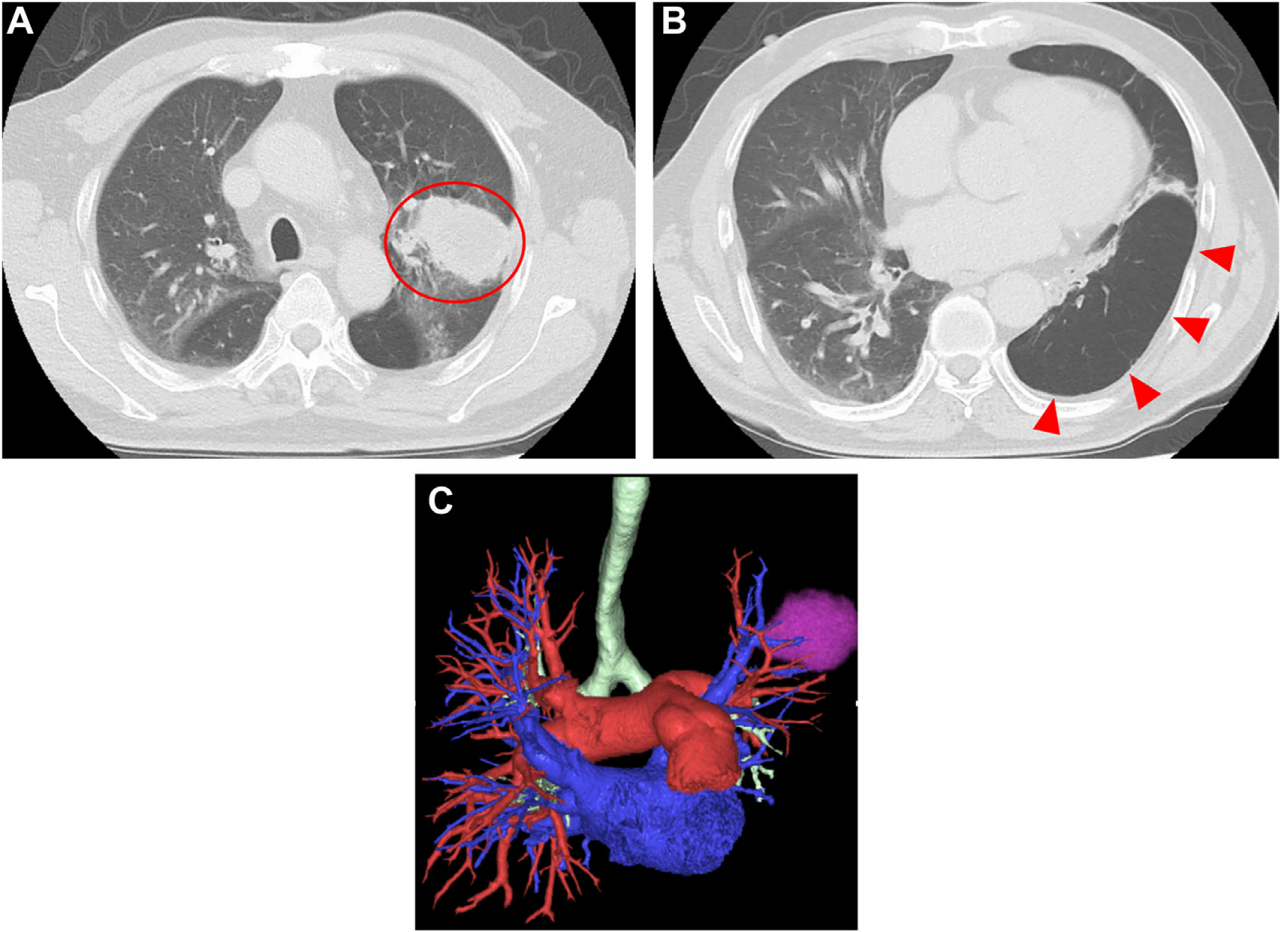
(A) Chest computed tomographic image shows an irregular mass (red circle) measuring 53 mm in diameter in the left upper lobe. (B) Hyperlucency and diminished vascularity in the left lower lobe (red triangles). (C) Three-dimensional computed tomographic image shows hypoplasia of the left pulmonary artery (A6-10) and inferior pulmonary vein.

A fluorine-18 fluorodeoxyglucose–positron emission tomography scan showed abnormal accumulation of fluorodeoxyglucose (maximum standardized uptake value of 19.54) in the tumor.

A pulmonary ventilation/perfusion scan revealed markedly decreased pulmonary blood flow in both the left upper lobe with the tumor and the left lower lobe with both hyperlucency and diminished vascularity. The right-to-left ratio of blood flow was 86.18% on the right and 13.28% on the left (Figure 3).

**Figure 3:**
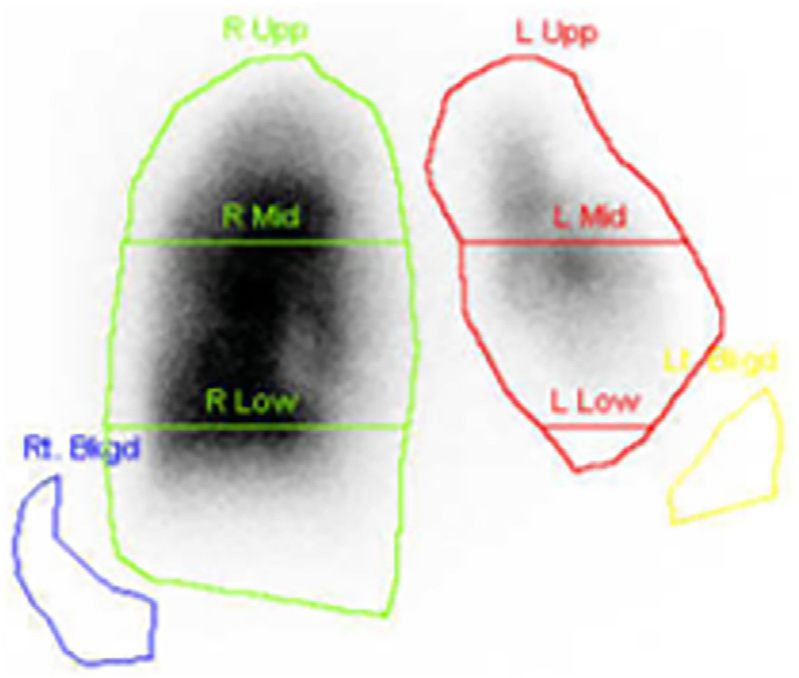
Pulmonary ventilation/perfusion scan shows markedly decreased pulmonary blood flow in both the left upper lobe with a tumor and in the left lower lobe with hyperlucency and diminished vascularity. (L, left; Low, lower; Mid, middle; R, right; Upp, upper.)

The clinical stage was cT3 N0 M0, stage IIB squamous cell lung carcinoma. In addition, SJMS was diagnosed for the left lower lobe. Robot-assisted thoracoscopic left pneumonectomy was performed. He had an uneventful course after the operation and was discharged home.

The postoperative pathology was squamous cell carcinoma with an invasion diameter of 45 × 45 × 30 mm. No lymph node metastasis was observed. The pathologic stage was pT2b N0 M0, stage IIA.

## Comment

The prevalence of SJMS is reported to be 0.01%, with most cases occurring in children and only a few cases in adults.^5^ Bronchiectasis is not a finding in all SJMS patients, but it is present in 30% of patients and affects clinical presentation and prognosis.^6^

SJMS as assessed radiologically is predominantly unilateral, and pathologic criteria to diagnose it have not yet established, so other causes of a hyperlucent lung, such as pneumothorax, asymmetric emphysema, congenital lobar emphysema, pulmonary artery hypoplasia, pulmonary embolism, and bronchial obstruction due to foreign body or mucus plugs, should be ruled out.^7^

Even though the pathogenesis of SJMS has not yet been completely understood, the syndrome has been reported to usually occur after lung infection early in childhood, possibly due to infective agents that damage the bronchioles and prevent normal development of the alveolar ducts. Bronchiolitis obliterans results in inflammation and fibrosis in the walls and contiguous tissues of the membranous and respiratory bronchioles, with narrowing of their lumens. Fibrosis of the alveolar septum leads to obstruction of the pulmonary capillary bed, reducing blood flow to the major pulmonary arterial segments, leading to secondary pulmonary vascular hypoplasia and bronchial dysplasia, and causing air trapping due to peripheral airway obstruction, peripheral lung hyperinflation, and emphysematous changes.^7^ This case of SJMS was concomitant with ipsilateral lung cancer. Although, concomitant SJMS and lung cancer is rare, we think that this may be because SJMS itself is a rare disease.

Preoperative CT showed hyperlucency of the left lower lobe with hypoplasia of the pulmonary vasculature, consistent with imaging findings characteristic of the syndrome. There were also repeated episodes of respiratory infections in childhood, although details are unknown, which may have been related to the onset of the disease.

Although the patient had already been receiving inhalation therapy for chronic obstructive pulmonary disease, he had been asymptomatic without respiratory distress or oxygen demand until he was diagnosed with SJMS by preoperative CT. Surgical intervention is considered in cases involving repeated infections or progressive dyspnea, but treatment of SJMS is typically conservative and supportive, including inhaled bronchodilators, low-dose inhaled steroid, respiratory physiotherapy, and infection control.^3^^,^^7^ There are hardly any reports of surgical intervention for SJMS; however, a few reports have suggested that resection of lesions is effective in improving pulmonary function and relieving symptoms of respiratory distress due to decrease in ventilation/perfusion mismatch in the affected lobe.^8^

In this case, CT showed pulmonary vascular hypoplasia in the left lower lobe, and respiratory function tests and pulmonary ventilation/perfusion scan showed significant pulmonary hypofunction in the left lung due to tumor in the upper lobe and SJMS in the lower lobe, so left pneumonectomy was planned for this patient.

Although intraoperative adhesions was anticipated, robot-assisted thoracoscopic surgery was selected because we thought minimally invasive surgery was best, considering pulmonary hypofunction and complications such as diabetes mellitus. Preoperative imaging showed that the pulmonary artery might be friable, but intraoperative findings showed that the pulmonary artery and other tissues were not friable. The left lower lobe was emphysematous, and the inferior pulmonary vessels were hypoplastic, but the left pneumonectomy was successful without any problems. The patient's postoperative course was uneventful, and he is still alive 8 months after surgery, after undergoing postoperative adjuvant chemotherapy, without recurrence.
